# Wispy, dystrophic hair in a pediatric patient: Expanding the differential with tricho-dento-osseus syndrome

**DOI:** 10.1016/j.jdcr.2025.08.015

**Published:** 2025-08-28

**Authors:** Michelle Chernyak, Brynn Sargent, Neda Zadeh, Bonnie A. Lee

**Affiliations:** a Department of Dermatology, University of California, Irvine, California; b Genetics Center, Orange, California

**Keywords:** dystrophic hair, ectodermal dysplasia, tricho-dento-osseous syndrome

## Introduction

Tricho-dento-osseous syndrome (Online Mendelian Inheritance in Man (OMIM):#190320; TDO) is an exceedingly rare autosomal dominant, completely penetrant genetic condition caused by heterozygous pathogenic variants in the distal-less homeobox 3 (DLX3) gene (OMIM:∗600525), which encodes a transcription factor crucial in fetal limb development.^1^^,^^2^ This leads to impaired epithelial-to-mesenchymal interactions and variable phenotypic defects in hair, teeth, and bone development.^1^^,^^2^ Here, we present a rare case of TDO to raise clinical awareness and increase diagnostic suspicion when encountering pediatric patients with dystrophic hair. Early identification of this condition is crucial to differentiate TDO from other similarly presenting conditions that may carry greater morbidity and mortality and to ensure that patients with TDO receive appropriate medical management including subspecialty care.

## Case report

A 20-month-old woman of Hispanic descent was brought to the clinic for evaluation of sparse hair growth. Unlike her siblings, the patient was born hairless and had minimal hair growth since birth, never requiring a haircut. Her mother reported increased nail fragility and microdontia of primary teeth with edentulism. Developmentally, she exhibited speech delay despite normal auditory evaluations. She subsequently qualified for and received speech therapy. At 20 months, she had a 3 to 5-word vocabulary and was also using sign language to communicate. She was physically active with appropriate gross motor development, had normal sweating ability, and had no increased frequency or severity of infections compared with her peers. The patient was evaluated by pediatric dentistry, who referred the patient for medical genetic testing because of concern for possible ectodermal dysplasia given her unusual dental morphology, although genetic results were still pending at the time of her initial presentation to dermatology.

**Fig 1:**
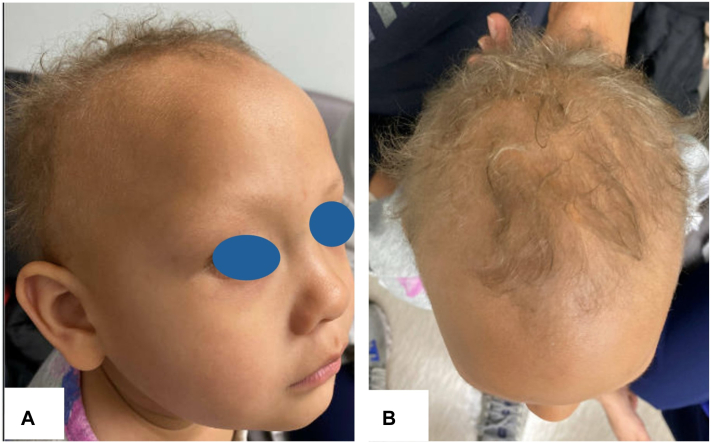
Nearly absent eyebrows and short, blonde, wispy scalp hair with an elevated anterior hairline (**A**) and a prominent widow’s peak (**B**).

Physical examination revealed light olive-colored skin consistent with that of her family background and was remarkable for admixed blonde and gray fine, curly hair sparsely scattered throughout the more posterior scalp (Fig 1), as well as sparse eyebrows and eyelashes. Fingernail and toenail growth appeared normal. Intraoral examination revealed missing upper frontal incisors and yellow discoloration of miniaturized lower frontal incisors without any conical teeth. Trichoscopy showed no other abnormalities, and a hair-pull test was negative. No other skin or joint abnormalities were appreciated. Light microscopy of multiple hair shafts demonstrated a mixture of lighter and darker hair shafts with relatively normal and evenly distributed melanin with foci of subtle melanin clumping.

Given the lightness of the hair color, the differential diagnosis included Chediak-Higashi syndrome and Griscelli syndrome, among others. Ectodermal dysplasia and other genetic conditions, including Menkes disease, trichothiodystrophy (TTD), and neonatal hypothyroidism, were all considered because of the paucity of hair and short, curly hair quality. The patient was referred to pediatric endocrinology and a subsequent extensive laboratory workup was notable only for mildly decreased free T4 with normal thyroid-stimulating hormone. comprehensive metabolic panel, complete blood count, erythrocyte sedimentation rate, and urinalysis, as well as several other nutritional laboratory tests, were unremarkable. Because the patient’s symptoms were congenital and the nutritional workup was unrevealing, the patient was referred for further medical genetic evaluation.

Genetics recommended and arranged a multigene-ectodermal dysplasia panel as an initial test, which revealed an incidental finding of a heterozygous pathogenic variant in the GJB2 gene (OMIM∗121011) consistent with a positive carrier status for autosomal recessive nonsyndromic hearing loss (DFNB1A;OMIM#220290). Whole-exome sequencing trio was performed and was diagnostic of TDO, with the detection of a heterozygous de novo likely pathogenic variant in the DLX3 gene consisting of c.534G>C (p.Gln178His).

## Discussion

First described in 1971 by Lichtenstein et al^3^ TDO is a rare, autosomal dominant completely penetrant genetic condition with associated abnormalities of the hair, teeth, and bone.^1^^,^^4^ The incidence is unknown and there have been a limited number of patient-reported cases that appear to be mostly isolated to a few families globally.^2^

Genetically, TDO is associated with heterozygous pathogenic variants in the DLX3 gene which encodes a transcription factor that plays a crucial role in fetal head and distal limb development.^1^^,^^2^ This protein plays an important role in regulating the development of bone, teeth, and hair.^5^ To date, approximately 13 specific pathogenic variants within the DLX3 gene have been described in association with TDO.^2^^,^^6^ There is wide phenotypic heterogeneity in the clinical manifestations of this condition; however, the most common phenotype includes enamel defects and taurodontism as the most reliable findings to delineate the diagnosis.^7^ Abnormalities in hair, nails, and bone are not always consistent.^5^

Our patient’s particular variant in DLX3: c.534G>C (p.Gln178His) has been described in available genome databases such as ClinVar with classifications including both “pathogenic” and “likely pathogenic” designations given by 2 separate laboratories. This aberration is located within the coding exon 3—a highly conserved homeobox region that encodes the homeodomain of the DLX3 protein.^2^ This specific variant has also been reported previously in the medical literature in 2 unrelated families of Chinese descent.^2^^,^^5^ In 2022, Liu et al^5^ described a 5-generation family with this pathogenic variant observed over 3 generations in an autosomal dominant inheritance pattern. This same author group had described another unrelated family from a similar global region with the same DLX3 variant and phenotype.^2^ Both manuscripts concluded that this specific DLX3 variant is likely responsible for TDO in the Han Chinese population.^2^^,^^5^ Interestingly, our patient was the simplex case for her family and is of Hispanic or Mexican descent, indicating that the c.553A>G variant is not exclusive to a particular population.

Clinically, dentition issues pose the greatest challenge to patients with TDO who may have difficulties with functional feeding (chewing ability), resulting in poor growth and failure to thrive. Other patients with TDO may first present with bone sclerosis, primarily involving the base of the skull, mastoids, and long bones.^8^ The kinky, curly hair found at birth in patients with TDO may sometimes lengthen with age and straighten by the second or third decade of life, although the mechanism for this change remains unclear.^5^^,^^8^

There are several pediatric conditions resulting in dystrophic or otherwise short, brittle, or kinky hair that may initially present with other nonspecific clinical features. Distinguishing these conditions is vital to ensure patients are referred to the appropriate subspecialists and parents are appropriately educated on their child’s prognosis and anticipated care. For example, TTD is associated with cognitive impairment, growth restriction, and recurrent severe infections of the gastrointestinal and respiratory tracts, whereas Menkes disease presents with neurologic abnormalities including seizures and hypotonia.^9^

Thorough physical and microscopic hair examination may help distinguish differential etiologies in pediatric patients who present with steely, kinky, sparse short hair as a cardinal feature. Polarized microscopy of hair shafts in TTD demonstrates alternating light and dark bands termed “tiger tail” banding.^10^ Hair microscopy in Menkes disease demonstrates characteristic pili torti with flattened hair shafts twisting 180° along the long axis in irregular intervals.^9^ Li et al^2^ commented on the presence of light-colored hair in individuals of the studied family as this was atypical of individuals of their particular background. Although TDO may not always exhibit pathognomonic microscopic features, patients with kinky, lightly pigmented hair, longitudinal grooves and scales along the hair shaft, and enamel abnormalities should be evaluated for TDO and considered for DLX3 molecular testing.

Distinguishing TDO from other ectodermal dysplasia etiologies during early childhood is important to adequately address possible comorbidities and provide accurate medical management during childhood including involvement of pediatric dentistry, along with pediatric nutritional services to avoid failure to thrive. It is also important to recognize that TDO is not limited to a particular population or ethnicity.

## Conflicts of interest

None disclosed.
